# Results from the WHO external quality assessment for the respiratory syncytial virus pilot, 2016‐17

**DOI:** 10.1111/irv.12771

**Published:** 2020-07-30

**Authors:** Sandra Jackson, Teresa C. T. Peret, Thedi T. Ziegler, Natalie J. Thornburg, Terry Besselaar, Shobha Broor, Ian Barr, Elsa Baumeister, Mandeep Chadha, Malinee Chittaganpitch, Badarch Darmaa, Joanna Ellis, Rodrigo Fasce, Belinda Herring, Kadjo Herve, Siddhivinayak Hirve, Yan Li, Maria Pisareva, Ann Moen, Amel Naguib, Rakhee Palekar, Varsha Potdar, Marilda Siqueira, Florette Treurnicht, Almiro Tivane, Marietjie Venter, Niteen Wairagkar, Maria Zambon, Wenqing Zhang

**Affiliations:** ^1^ Global Influenza Programme World Health Organization Geneva Switzerland; ^2^ Division of Viral Diseases, Respiratory Viruses Branch Centers for Disease Control and Prevention (CDC) Atlanta GA USA; ^3^ Research Center for Child Psychiatry University of Turku Turku Finland; ^4^ Medicine and Health Sciences Shree Guru Gobind Singh Tricentenaryl University Gurugram India; ^5^ Victorian Infectious Diseases Reference Laboratory Peter Doherty Institute for Infection and Immunity Melbourne VIC Australia; ^6^ Departamento Virologia INEI‐ANLIS Carlos G Malbrán Buenos Aires Argentina; ^7^ National Institute of Virology Indian Council of Medical Research Pune India; ^8^ Department of Medical Sciences Ministry of Public Health Nonthaburi Thailand; ^9^ Virology Laboratory National Center for Communicable Diseases Ulanbaatar Mongolia; ^10^ Virus Reference Department Public Health England London UK; ^11^ Sub‐department of Viral Diseases Instituto de Salud Pública de Chile Santiago Chile; ^12^ African Region Office World Health Organization Brazzaville Republic of Congo; ^13^ Department of Epidemic Viruses Institut Pasteur de Côte d’Ivoire Abidjan Côte d’Ivoire; ^14^ Influenza and Respiratory Viruses Section National Microbiology Laboratory Public Health Agency of Canada Winnipeg MB Canada; ^15^ Laboratory of Molecular Virology Smorodintsev Research Institute of Influenza St. Petersburg Russian Federation; ^16^ Central Public Health Laboratory Ministry of Health Cairo Egypt; ^17^ Pan American Health Organization Washington DC USA; ^18^ Institute Oswaldo Cruz/Fiocruz Rio de Janerio Brazil; ^19^ Centre for Respiratory Diseases and Meningitis National Institute for Communicable Diseases Johannesburg South Africa; ^20^ Laboratório de Isolamento Viral Instituto Nacional de Saúde Maputo Mozambique; ^21^ Center for Viral Zoonosis, Department of Medical Virology University of Pretoria Pretoria South Africa; ^22^ Bill and Melinda Gates Foundation Seattle WA USA

**Keywords:** RSV external quality assessment, polymerase chain reaction, respiratory syncytial viruses

## Abstract

**Background:**

External quality assessments (EQAs) for the molecular detection of respiratory syncytial virus (RSV) are necessary to ensure the provision of reliable and accurate results. One of the objectives of the pilot of the World Health Organization (WHO) Global RSV Surveillance, 2016‐2017, was to evaluate and standardize RSV molecular tests used by participating countries. This paper describes the first WHO RSV EQA for the molecular detection of RSV.

**Methods:**

The WHO implemented the pilot of Global RSV Surveillance based on the WHO Global Influenza Surveillance and Response System (GISRS) from 2016 to 2018 in 14 countries. To ensure standardization of tests, 13 participating laboratories were required to complete a 12 panel RSV EQA prepared and distributed by the Centers for Disease Control and Prevention (CDC), USA. The 14th laboratory joined the pilot late and participated in a separate EQA. Laboratories evaluated a RSV rRT‐PCR assay developed by CDC and compared where applicable, other Laboratory Developed Tests (LDTs) or commercial assays already in use at their laboratories.

**Results:**

Laboratories performed well using the CDC RSV rRT‐PCR in comparison with LDTs and commercial assays. Using the CDC assay, 11 of 13 laboratories reported correct results. Two laboratories each reported one false‐positive finding. Of the laboratories using LDTs or commercial assays, results as assessed by Ct values were 100% correct for 1/5 (20%). With corrective actions, all laboratories achieved satisfactory outputs.

**Conclusions:**

These findings indicate that reliable results can be expected from this pilot. Continued participation in EQAs for the molecular detection of RSV is recommended.

## 1 INTRODUCTION

The World Health Organization (WHO) Global Influenza Program (GIP) launched a pilot in 2016 to build global respiratory syncytial virus (RSV) surveillance on the well‐established WHO Global Influenza Surveillance and Response System (GISRS).^1^ RSV is a significant public health problem, causing an estimated annual 33 million acute lower respiratory infections (ARI) in children under 5 years.^2^ Very young children and the elderly are at risk of experiencing severe RSV infections.^3^, ^4^ Particularly in low‐income countries, RSV in infants causes considerable mortality, the magnitude of which is not be fully understood.^5^ Although monoclonal antibodies as palivizumab have existed for some time, there are several RSV vaccines and monoclonal antibodies in phase I, II, and III clinical trials. Therefore, new RSV interventions may be on the market within the next 5‐10 years.^6^

The establishment of a WHO Global RSV Surveillance is essential to support countries’ understanding of RSV epidemiology in different regions and associated disease burden and is instrumental to understand the global circulation of different strains of RSV. A key objective of the WHO Global RSV Surveillance is to standardize RSV molecular detection and surveillance using the already established GISRS platform.

Although several commercial and RSV LDTs are available for RSV detection, there are no agreed reference standards for RSV molecular testing and ongoing evaluations of performance through external quality assessments (EQAs)^7^, ^8^, ^9^, ^10^ Fourteen countries from all 6 WHO regions where National Influenza Centers (NIC) have demonstrated reliable quality of molecular detection of influenza viruses through the annual influenza WHO EQA, were selected to participate in the pilot. In addition, three laboratories with long‐standing experience in RSV detection and research agreed to serve as RSV Reference Laboratories, primarily to provide technical support to the 14 pilot laboratories.^11^

An established real‐time (r) RT‐PCR was provided to the pilot participant laboratories as the standard assay for the RSV detection by the CDC, USA.^12^ Pilot laboratories were given the option to use LDTs or commercial real‐time RT‐PCR assays following validation against the CDC RSV rRT‐PCR assay.^12^ This paper describes and summarizes the findings of the WHO RSV EQA that was launched in 2016 to 2017, prior to the implementation of RSV surveillance in participating countries.

## 2 METHODS

### 2.1 Selection of pilot countries

Two or three countries with laboratories designated as NICs and with proven molecular technical expertise in influenza diagnostics along with other demonstrated influenza surveillance capabilities were selected from each of the six WHO Regions. Thirteen countries were initially selected and received the EQA panel prepared and distributed by the CDC USA. The 14th country joined the pilot later in that year and participated in the RSV EQA panel provided by Quality Control for Molecular Diagnostics (QCMD). This QCMD panel 2016 was also validated by CDC. Details of the QCMD EQA performance by the 14th country are omitted from this analysis. Some laboratories in these countries were already performing RSV testing using various LDTs or commercial molecular assays. Participating countries included Mozambique, Sierra Leone, and South Africa in the African Region; Argentina, Brazil, Canada, and Chile in the American Region; Egypt in the Eastern Mediterranean Region; Russia and the United Kingdom in the European Region; India and Thailand in the South‐East Asian Region; and Australia and Mongolia in the Western Pacific Region.

### 2.2 Selection of RSV reference laboratories and participation in the QCMD EQA

Three laboratories (Respiratory Viruses Branch, Division of Viral Diseases, Centers for Disease Control and Prevention (CDC), United States; Public Health England, United Kingdom of Great Britain and Northern Ireland; and the National Institute of Communicable Diseases, South Africa participated in the QCMDEQA for RSV.

These laboratories had established RSV technical expertise and molecular detection experience and were invited to function as RSV Reference Laboratories to support the 14 pilot countries. Reference laboratories were required to first participate and perform suscessfully in an RSV EQA provided by QCMD, Glasgow, Scotland. All laboratories successfully achieved a 100% correct score.^13^ RSV Reference Laboratories functioned according to Terms of Reference developed by the WHO Global Influenza Program and the WHO Global RSV Surveillance.^13^, ^14^

### 2.3 CDC RSV rRT‐PCR assay

The CDC RSV rRT‐PCR assay was selected as the reference assay for the WHO RSV Surveillance pilot. The assay included primers and probes targeting conserved regions of the RSV matrix gene and a housekeeping human RNAse P (RNP) gene that were synthesized at the CDC Core Facility and distributed to the participant laboratories. Prior to receiving the EQA panel, each laboratory received a detailed CDC protocol with a list of approved commercial nucleic acid extraction methods, real‐time RT‐PCR kits and platforms for laboratory guidance. The CDC RSV rRT‐PCR was validated with the following Real‐Time PCR instruments: 7500 Fast Dx Real‐Time PCR and ViiA 7 Real‐Time PCR Systems (Applied Biosystems); Mx3000P and Mx3005P QPCR System (Agilent Technologies); and iCycler IQ5 and CFX96 (Bio‐Rad Laboratories).

Extraction systems assessed with successful outputs with this assay included the following: QIAamp^®^ MinElute Virus and Viral RNA Mini Spin Kits (QIAGEN); NucliSENS^®^ EasyMag^®^ and miniMag^®^ (bioMérieux); MagMAX^™^ Express and Total Nucleic Acid Isolation Kit (Life Technologies); and MagNA Pure Compact System and Nucleic Acid Isolation Kit 1 (Roche Applied Science). The RNA extraction kits and PCR kits were provided through the CDC International Reagent Resource (IRR).

### 2.4 Preparation and composition of the CDC RSV EQA panel

Each CDC panel consisted of 20 freeze‐dried samples prepared from RSV‐infected or uninfected cell cultures (Table 1). Four RSV strains were used 15353_ON1 (RSV A) ^15^, ^16^ RSV Long strain (RSV A),^17^ 53530_BA (RSV B),^18^ and 209_GB3 (RSV B).^19^, ^20^ Viruses were grown using Hep‐2 monolayer with 10% MEM + 20 mM HEPES and inactivated by gamma‐irradiation. Replicate 10‐ and 2‐fold dilutions of RSV strains Long, 209_GB3, 15353_ON1 and 53530_BA were performed in viral transport media (VTM) and frozen at −70°C. Three replicates of each dilution of each strain were thawed, and total nucleic acid was extracted (NucliSens easyMAG, BioMerieux, USA) and tested using the CDC rRT‐PCR assay. Three dilutions for each RSV strain representing high, medium, and low virus loads were selected to represent the range of Ct values typically found in children and adult clinical respiratory specimens.^21^ RSV A Long and 15353_ON1 sample series were set from high to low Ct, and RSV B 209_GB3 and 53530_BA sample series were set from low to high Ct values. Four negative samples were included in the test panel (samples 4, 9, 14, and 19). In order to assess reproducibility of test results, 4 of the RSV‐positive samples were included as duplicates (samples 2 and 8, 7 and 13, 11 and 18, and 15 and 20).

**Table 1:** WHO RSV External Quality Assessment (EQA), 2016, for pilot laboratories’ performance in the molecular detection of RSV: Panel composition, expected and reported results

RSV	Sample number	Duplicate samples	Expected Ct values		Expected result	Reported Ct values		Final EQA Results of 3 Laboratories scoring < 100% (N = 3/13)					
Group_Strain Name						Mean (range)	Mean (range)	Laboratory 6		Laboratory 10		Laboratory 13	
			RSV	RNP		CDC rRT‐PCR	Commercial/LDT PCR	CDC rRT‐PCR	Commercial/LDT PCR	CDC rRT‐PCR	Commercial/LDT PCR	CDC rRT‐PCR	Commercial/LDT PCR
A_Strain Long	1		≤32	≤33	RSV Pos	31.26 (27.2‐35.8)	31.46 (29.0‐31.5)	Correct	NP	Correct	Correct	Correct	Correct
A_Strain Long	2	2 = 8	≤30	≤34	RSV Pos	28.38 (27.5‐31.5)	29.36 (25.7‐33.0)	Correct	NP	Correct	Correct	Correct	Correct
A_Strain Long	3		≤26	≤34	RSV Pos	23.38 (22.1‐26.6)	24.34 (21.5‐28.0)	Correct	NP	Correct	Correct	Correct	Correct
Blank	4		Neg	≤33	RSV Neg	NA	NA	Correct	NP	Correct	Correct	Correct	Correct
B_Strain 209_GB3	5		≤28	≤31	RSV Pos	27.71 (25.1‐31.1)	28.24 (20.1‐32.2)	Correct	NP	Correct	Correct	Correct	Correct
B_Strain 209_GB3	6		≤32	≤29	RSV Pos	32.18 (28.6‐34.1)	34.73 (34.0‐35.4)	Correct	NP	Correct	Correct	Correct	Incorrect^c^
B_Strain 209_GB3	7	7 = 13	≤35	≤33	RSV Pos	33.25 (27.5‐35.8)	36.00 (35.0‐37.0)	Correct	NP	Correct	Correct	Correct	Incorrect^c^
A_Strain Long	8	8 = 2	≤30	≤34	RSV Pos	29.12 (27.1‐31.3)	29.98 (29.0‐ 33.0)	Correct	NP	Correct	Correct	Correct	Correct
Blank	9		Neg	≤33	RSV Neg	NA	NA	Correct	NP	Incorrect ^b^	Incorrect ^b^	Correct	Correct
A_Strain 15353_ON1	10		≤37	≤33	RSV Pos	34.36 (29.1‐37.1)	35.78 (27.9‐41.4)	Correct	NP	Correct	Correct	Correct	Correct
A_Strain 15353_ON1	11	11 = 18	≤29	≤33	RSV Pos	27.52 (23.1‐30.0)	35.78 (27.9‐41.4)	Correct	NP	Correct	Correct	Correct	Correct
A_Strain 15353_ON1	12		≤27	≤33	RSV Pos	24.52 (22.1‐28.2)	25.34 (20.9‐28.0)	Correct	NP	Correct	Correct	Correct	Correct
B_Strain 209_GB3	13	13 = 7	≤35	≤33	RSV Pos	32.31 (28.0‐35.1)	25.34 (20.9‐28.0)	Correct	NP	Correct	Correct	Correct	Incorrect^c^
Blank	14		Neg	≤33	RSV Neg	NA	NA	Incorrect ^a^	NP	Incorrect ^b^	Incorrect ^b^	Correct	Correct
B_Strain 53530_BA	15	15 = 20	≤26	≤31	RSV Pos	24.09 (21.0‐27.5)	25.82 (18.8‐29.8)	Correct	NP	Correct	Correct	Correct	Correct
B_Strain 53530_BA	16		≤28	≤31	RSV Pos	28.48 (24.5‐31.3)	29.12 (23.1‐33.0)	Correct	NP	Correct	Correct	Correct	Correct
B_Strain 53530_BA	17		≤34	≤32	RSV Pos	31.82 (24.2‐37.4)	32.70 (26.9‐36.3)	Correct	NP	Correct	Correct	Correct	Correct
A_Strain 15353_ON1	18	18 = 11	≤ 29	≤ 33	RSV Pos	26.88 (23.3‐30.0)	28.40 (25.4‐29.9)	Correct	NP	Correct	Correct	Correct	Correct
Blank	19		Neg	≤34	RSV Neg	NA	NA	Correct	NP	Correct	Correct	Correct	Correct
B_Strain 53530_BA	20	20 = 15	≤26	≤31	RSV Pos	24.33 (23.0‐26.2)	26.14 (20.9‐28.4)	Correct	NP	Correct	Correct	Correct	Correct

Abbreviations

Pos, positive; NA, not applicable; Neg, negative; NP, not performed.

^a^ Reported as positive.

^b^ Reported as inconclusive.

^c^ Reported as negative.

### 2.5 Distribution of RSV EQA panel to participating laboratories

RSV EQA panels were dispatched at ambient temperature by courier service between December 2016 and March 2017 by CDC. The RSV rRT‐PCR kits were shipped on dry ice. Laboratories were requested to return their results within 4 weeks after receiving the materials. Upon shipping, each laboratory received a message with a unique number, a copy of the protocols and package inserts and a fillable result form. The unique assigned number was used for further communications with each laboratory. Laboratories reported results electronically to CDC for analysis using unique assigned numbers, safeguarding participant laboratories confidentiality. Following analysis, CDC reported results to participating laboratories and to WHO maintaining confidentiality.

### 2.6 Other molecular methods for RSV detection

Some pilot laboratories evaluated their LDTs or commercially available rRT‐PCR assays for the detection of RSV. If these tests performed satisfactorily with the CDC RSV EQA panel, these laboratories could use these assays for the duration of the pilot. For laboratories that did not return correct results, review of methodologies and corrective actions were taken under the guidance of CDC RSV Reference Laboratories. Where necessary, laboratories were recommended to use the CDC rRT‐PCR assay.

## 3 RESULTS

Using the CDC RSV assay, 11 of the 13 laboratories returned correct EQA final results from all 20 specimens within the expected time frame of four weeks after receiving the EQA panel. Late results (results submitted after 4 weeks) were associated with handling issues by the country customs. The validity of the delayed panel was verified by running an identical panel at the CDC laboratory under similar conditions. The outputs were found to correlate and to be satisfactory. Of the 13 laboratories, one laboratory (laboratory 6) reported a single false positive which was associated with the use of a PCR platform which had not been previously validated for use with the CDC RSV assay. Laboratories which used both CDC and LDTs or commercial assays included the laboratories 5, 7, 8, 10, and 13. Laboratory 5 scored 100% correct with Ct readings for both the CDC and the commercial assay correlating well with expected values. Laboratory 10 reported two false‐positive results using the CDC and a LDT assays (Table 1). Laboratory 13 obtained 3 false‐negative results for specimens containing lower viral loads of RSV B_Strain 209_GB3 with a commercial assay but correctly identified RSV B_Strain 209_GB3 with the CDC assay (Table 1). However, using this commercial assay, laboratory 13 correctly identified RSV A_Strain 15353_ON1, RSV A_Strain Long, and RSV B_Strain 53530_BA in 10 of the 13 specimens and yielded lower Ct values than that of the CDC RSV assay.

Although laboratories 7 and 8 reported correct final EQA results (based on readings of the CDC assay), the actual Ct readings using the commercial assay were out of range for the expected results. Laboratory 7 reported Ct readings of 37.5 for specimen 13 (expected Ct:<35 for RSV B_Strain 209_GB3), 31.9 for specimen 16 (expected Ct <28 for RSV B_Strain 53530_BA), and 36 for specimen 17 (expected Ct <34   for RSV B_Strain 53530_BA). However, for specimen 15 using the commercial assay, laboratory 7 reported a Ct of 26.6 which approximated the expected Ct of <26 for RSV B_Strain 53530_BA. The corresponding Ct values reported by laboratory 7 using the CDC assay were 34.3 for specimen 13 (RSV‐ B_Strain 209_GB3), 29.5 for specimen 16 (RSV B_Strain 53530_BA), and 37.4 for specimen 17 (RSV B_Strain 53530_BA).

Laboratory 8 also reported results for both the CDC and a commercial assay. The final EQA result was reported correctly as positive; however, the actual Ct reading using the commercial assay was out for range for specimen 10 (RSV A_Strain 15353_ON1, expected Ct <37) and read as 41.4 for the commercial assay and 34.8 for the CDC assay. On average, the Ct values reported as obtained with the CDC RSV assay matched well with the expected Ct values established by the CDC RSV Reference Laboratory through repeated testing. While the mean Ct values reported by the 13 pilot laboratories were within less than one cycle, a cycle difference of 2 or more was reported in 13 cases. Using both the CDC assay and a LDT, laboratory 14 scored 100% with the QCMD panel which was also validated by the CDC.

## 4 DISCUSSION

Thirteen of 14 laboratories participated in a RSV EQA conducted by the WHO GIP and provided by the RSV Reference Laboratory for the Pilot, the Respiratory Viruses Branch of CDC, USA. The EQA panel consisted of 16 RSV‐positive and 4 RSV‐negative samples dispatched to the pilot laboratories in late 2016 to early 2017. All 14 pilot laboratories received laboratory protocols and reagents for an RSV rRT PCR assay developed and validated by the Respiratory Viruses Branch of CDC, USA. Five of the 13 laboratories also evaluated LDTs or commercial assays already in use at their laboratories with the CDC RSV EQA.

Overall, the pilot laboratories performed well in this quality assessment. No false‐negative result was reported when using the CDC RSV assay. Two laboratories reported one false‐positive result. Of these, one laboratory (laboratory 6) used the CDC assay only and reported a false‐positive result for specimen 14. This may have resulted from contamination as the Ct value read at 32.45. The second laboratory (laboratory 10) evaluated both CDC and a LDT assays and reported incorrect results (reported as inconclusive and scored as false positive) for specimens 9 and 14 with both assays. The Ct values for specimens 9 and 14 were found to be >38, and these results should have been interpreted as negative. Although laboratories 7 and 8 reported correct final EQA results based on CDC assay readings, the Ct values were out of range for expected values and are therefore interpreted as incorrect for this analysis. Using the Ct reading of 41.4 for specimen 10 (RSV A_Strain 15353_ON1), laboratory 8 would have reported the result as a false negative based on the commercial assay. Using results of the commercial assay only, similarly laboratory 7 would have reported 3 false negatives or inconclusive results. Although the final EQA result for specimen 17 (expected Ct <34 for RSV B_Strain 53530_BA) was reported correctly as positive by laboratory 7 using the CDC assay, the Ct value of 37.4 was higher than expected. It is possible that extraction techniques or methods could account for variations in the quality of extracts.

Ct values reported for the 16 RSV‐positive specimens by the 13 laboratories varied but fell within expected range as established by CDC (Table 1). This variation in Ct values is most likely due to differences in PCR chemistries and equipment used by individual laboratories. Different shipping and storage conditions in the participating laboratories may also explain some of this deviation. Other possible contributing factors could have been different working conditions and varying technician skills. The fact that one laboratory reported the lowest Ct values and another laboratory the highest Ct values within the test mean range of the 16 RSV‐positive specimens may underscore this reasoning. With regard to the commercial assay used by laboratory 10, non‐conforming results from the lack of the sensitivity were observed with RSV B_Strain 209_GB3.

It is possible that the EQA specimens could have been handled with special care although such specimens should be processed according to standard laboratory practices. It is challenging to coordinate an EQA panel testing such that the EQA specimens should be blinded for the laboratory staff. Although not required, the CDC EQA samples, upon reconstitution, provided enough material for a second nucleic acid extraction and rRT‐PCR testing in anticipation of an unsuccessful first test.

The overall results presented during this exercise indicated that the pilot laboratories, particularly when using the recommended RSV assay and reagents provided by CDC, performed very well. Laboratories returned reasonably matched values for the duplicate specimens, and generally their findings corresponded well to the expected results. National Influenza Centers are regularly tested for their performance in the detection of different types and subtypes of influenza viruses. With a history of excellent performance in influenza EQA, it is therefore not surprising that the laboratories recruited for this RSV pilot also proved high accuracy in the molecular detection of RSV. This provides confidence that the RSV pilot could yield reliable results to be used in the analysis of clinical and epidemiological data.

Pilot laboratories used information regarding their performance, which was generated from the CDC RSV EQA panel to make appropriate changes and improvements in the laboratory detection of RSV.

Corrective action for one laboratory included that a change was made to use one of the real‐time PCR platforms and software versions which had been validated and recommended by CDC. This resulted in that laboratory achieving the expected output. Taking corrective actions and investigation of test non‐conformities, inconsistencies, and deficiencies provides an opportunity for standardized laboratory detection improvements. Although several types of laboratory tests are available for the diagnosis of RSV, it is necessary to have a standard reference protocol and assessments of performance by EQAs. Rapid diagnostic antigen detection tests although useful are generally reliable in young children but less useful in older children and adults. The relevance of EQAs continues to be important with the growing trend to use molecular point of care tests at emergency care instead of antigen tests. ^22^, ^23^ Highly sensitive RT‐PCR assays should be considered when testing adults, due to their ability to detect low viral loads in clinical specimens.

Although several RT‐PCR RSV LDTs and commercial assays are available, the sensitivity and specificity of these assays should be periodically evaluated against a reference standard to avoid the under‐ or over‐reporting of RSV infections. This pilot focused on the quality of detection of RSV and therefore used primers and probes to highly conserved regions in the RSV M gene.

Following satisfactory performance of laboratories in the EQA using either the CDC or non‐CDC assay, participating laboratories were given the option to conduct RSV surveillance using the assay of their choice. As CDC receives clinical specimens and RSV isolates from many different countries, they can closely monitor the evolution or RSV genes targeted by their assay and can alert participating laboratories should mutations in the targeted sequences affect the performance of the assay. It is unlikely however that only one reference assay will be used in ongoing RSV surveillance hence the necessity for EQAs. The participation of laboratories in RSV EQAs is critical to compare and evaluate the performance of various molecular assays. Furthermore, there is a danger in using one primer design, as when this fails all laboratories will fail.

## 5 CONCLUSIONS

The comparison of EQA results with a variety of assays is a good surrogate measure of performance as there are no international molecular standards available to date for the molecular detection of RSV. ^24^ The molecular detection of RSV was standardized through the CDC RSV Proficiency Panel in 13 pilot countries of the 6 WHO regions. Laboratories performed well using the CDC RSV molecular assay in comparison with LDTs and commercial assays. These countries were the first participants of the pilot of the WHO Global RSV Surveillance. With the expected introduction of RSV vaccines in the future and the need to provide accurate data to close knowledge gaps in the epidemiology of RSV, the standardization of molecular laboratory detection methods will continue to play a key role in surveillance. As the WHO RSV surveillance continues to expand, implementation of quality assessment methods for RSV A and B subgroup detection and genetic variability should play equally important roles.

## AUTHOR CONTRIBUTION

Jackson Sandra, Peret Teresa C. T., Ziegler Thedi, Besselaar Terry, Broor Shobha, Barr Ian, Baumeister Elsa, Thornburg Natalie J., Hirve Siddhivinayak, and Zhang Wenqing were part of the Writing Group and contributed to the analysis and writing of the manuscript. All authors contributed to the conceptualization and implementation of the World Health Organization global respiratory syncytial virus surveillance pilot and the implementation of the WHO RSV EQA. All authors critically reviewed and approved the manuscript.

## DISCLAIMER

The findings and conclusions in this report are those of the author(s) and do not necessarily represent the official position of the Centers for Disease Control and Prevention, USA, or the respective institutes of the authors. Names of specific vendors, manufacturers, or products are included for public health and informational purposes; inclusion does not imply endorsement of the vendors, manufacturers, or products by the Centers for Disease Control and Prevention or the USA Department of Health and Human Services. Siddhivinayak Hirve, Sandra Jackson, Belinda Herring, Rakhee Palekar, Ann Moen, and Wenqing Zhang work with the World Health Organization. The authors are responsible for the views expressed in this publication, and they do not necessarily represent the decisions, policy, or views of the World Health Organization.
